# Arteriovenous malformation of small intestine successfully treated by double-balloon enteroscopy and laparoscope-assisted surgery

**DOI:** 10.1093/jscr/rjac606

**Published:** 2022-12-30

**Authors:** Takayuki Tohma, Yasuyuki Okabe, Masaya Ushio, Masaya Saito

**Affiliations:** Department of Acute Care Surgery, Chiba Emergency Medical Center, Chiba, Japan; Life Support and Emergency Center, Nagaoka Red Cross Hospital, Niigata, Japan; Department of Acute Care Surgery, Chiba Emergency Medical Center, Chiba, Japan; Department of Gastroenterology and Hepatology, Seikei-Kai Chiba Medical Center, Chiba, Japan

## Abstract

Arteriovenous malformation (AVM) of the small bowel is a rare disease and can be sometimes difficult to treat due to the diagnostic difficulty. We herein report a case of small intestinal bleeding of AVM successfully treated with double-balloon enteroscopy (DBE) and laparoscope-assisted resection. A 44-year-old man complained of hematochezia and visited the previous doctor. He underwent gastroscopy and colonoscopy, but no bleeding site was detected. However, he rebled 2 days later and became hypotensive. Abdominal computed tomography revealed a hypervascular nodule in the jejunum. He was transferred to our institution for further treatment. DBE was performed and revealed a small pulsatile lesion with a tiny mucosal break. We then injected a marking tattoo. Two days later, he underwent an operation. We were able to easily locate the tattooed lesion laparoscopically and performed jejunal partial resection. His postoperative course was uneventful. DBE enabled a precise diagnosis and minimal invasive surgery.

## INTRODUCTION

Arteriovenous malformation (AVM) of the gastrointestinal tract is a rare disease [1]. However, it sometimes causes massive intestinal hemorrhaging. One of the most difficult problems is determining its location in the small intestine, as the small intestine cannot be easily explored using typical examinations, such as gastroscopy and colonoscopy.

We herein report a rare case of obscure gastrointestinal bleeding due to small intestinal AVM. We preoperatively detected its location using double-balloon enteroscopy (DBE) and successfully resected it by laparoscope-assisted surgery.

## CASE REPORT

A 44-year-old male patient complained of hematochezia and was hospitalized in another hospital. Gastroscopy and colonoscopy performed after admission showed no evidence of the bleeding site. Two days later, he suddenly bled again and became hypotensive. Abdominal contrast-enhanced computed tomography (CE-CT) revealed a hypervascular lesion in the jejunum. He was then transferred to our institution for further treatment.

**Figure 1:**
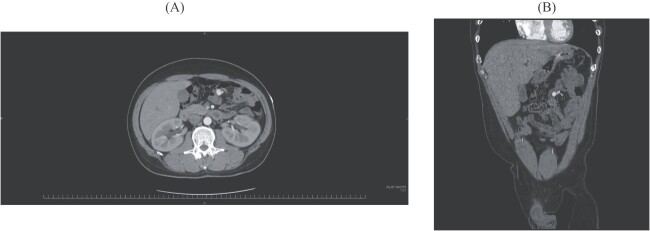
(**A**) Axial image of CE-CT shows a round, hypervascular nodule. (**B**) Coronal image shows vascular dilatation in the mesentery close to the nodule.

On admission, his vital signs were normal without evidence of active bleeding. He had no medical history. He had a hemoglobin of 8.5 g/dL. A physical examination demonstrated no particular findings in his abdomen. CE-CT obtained at the previous hospital showed a round shape nodule of 2 cm in size. It was accompanied by vascular dilatations in the jejunum, but there was no extravasation in the whole intestine (Fig. 1).

**Figure 2:**
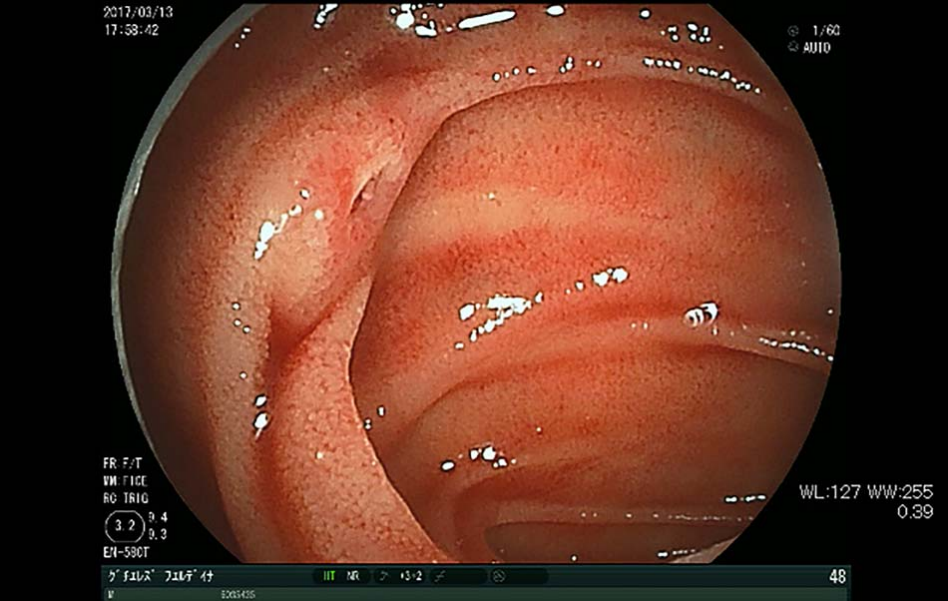
DBE shows a tiny mucosal break with pulsatile submucosal uplifting in the jejunum.

He needed surgery, but the lesion was considered too small to detect intraoperatively. We therefore decided to preoperatively perform DBE to determine its exact location. DBE showed fresh blood clots in the jejunum around 2 m distal from the ligament of Treitz with no bleeding site behind them. We found a smooth uplift with pulsation 10 cm distal from that point and identified a tiny mucosal break without active bleeding at the center of the lesion (Fig. 2). We performed tattooing and clipping around the lesion.

**Figure 3:**
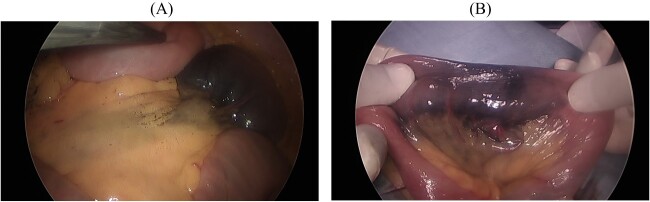
(**A**) Laparoscopy easily shows the tattooed jejunum. (**B**) Gross findings show dilated pulsatile vessels in the mesentery.

Two days after DBE, we performed an operation assisted by laparoscopy. We inserted the first port through the umbilicus and added two ports to both sides of his lower abdomen. We located the tattooed section of the intestine easily and removed it from his abdominal cavity through his umbilicus (Fig. 3). We then performed partial resection of the small intestine followed by reconstruction in a conventional hand-sewn manner.

**Figure 4:**
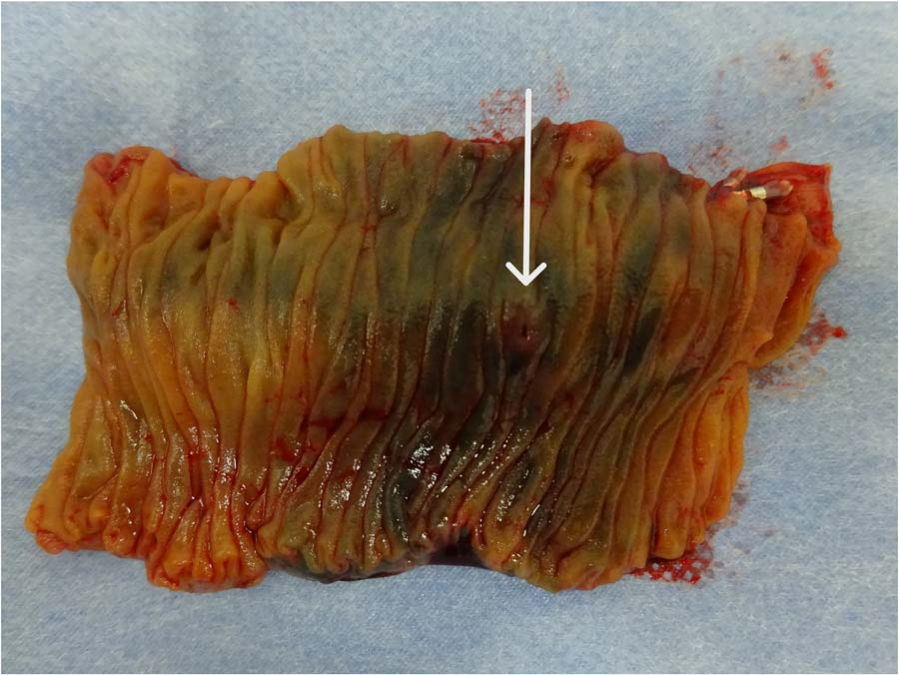
Gross findings of the resected specimen show no abnormal wall changes except for a tiny mucosal break in the jejunal mucosa (arrow).

**Figure 5:**
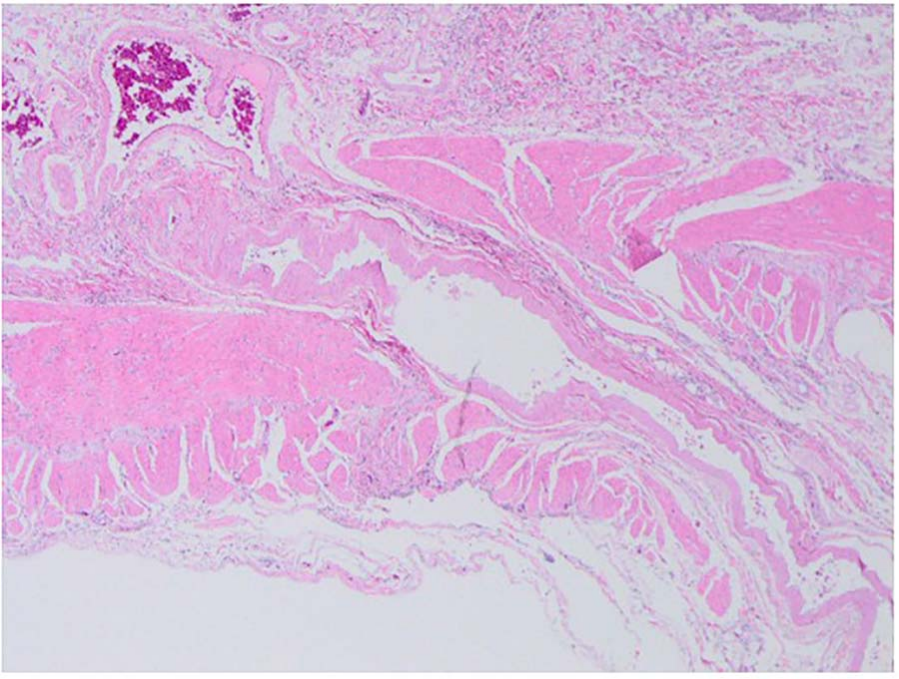
Histopathological findings show dilated arteries and veins from the submucosal layer to the mesentery.

His postoperative course was uneventful, and he was discharged from our hospital on Day 7 after surgery. A histopathological examination confirmed AVM without neoplastic changes (Figs 4 and 5).

## DISCUSSION

We herein report a rare case of obscure gastrointestinal bleeding due to small intestinal AVM. Preoperatively, we detected its location using DBE and successfully resected it by laparoscope-assisted surgery.

In our case, DBE was useful for diagnosing the lesion precisely, suggesting its utility in managing small intestinal AVMs. One of the most difficult problems in the management of small intestinal hemorrhaging is the confirmation of the bleeding site [1]. DBE has recently become available to be used in cases of small intestinal bleeding [2]. Endoscopic findings suggesting recent hemorrhaging, such as obvious bleeding, spurring bleeding, oozing and visible vessels, are diagnostic and definitive [3]. It is relatively easy to detect these findings in cases of mucosal lesions, such as ulcers, polyps and cancer. However, more careful observation is required to detect AVMs, as they are not derived from the mucosal layer and usually only have slight changes. Furthermore, AVMs of the small bowel are rare diseases, and their typical endoscopic findings remain unclear [4]. In general, AVMs are described as submucosal uplifting with redness, vascular proliferation and vascular ectasia. Hirakawa et al. reported that pulsatile submucosal uplifting accompanied by a small red patch on top might be an important finding indicating AVM [5]. In the present case, the lesion was detected as a pulsatile nodule without obvious mucosal findings which had been previously reported. It only had a tiny mucosal break, which might have been an important finding.

It is also suggested that a precise diagnosis using DBE can allow the selection of a less-invasive laparoscopic procedure. Reportedly, 5–37% of patients who undergo resection of AMVs experience rebleeding due to incomplete excision [6]. Once patients need surgery for the treatment of small intestine AVMs, it can be difficult to localize the lesions during the operation [7]. To overcome this problem, several intraoperative techniques have been reported, including preoperative metallic coil embolization [7], intraoperative selective angiography with indocyanine green injection [5, 8] methylene blue injection [6] and intraoperative surgical techniques, such as palpation and blind segmental resection, based on the preoperative diagnosis. In the present case, preoperative DBE enabled marking with clipping and submucosal injection of a tattoo for subsequent surgery [2]. Preoperative localization of the small bowel lesion enabled minimally invasive treatment. Regarding surgical intervention, laparoscopic surgery in small bowel resection has advantages over laparotomy, including the reduction of adhesions and incisional hernias [3]. In the present case, a laparoscopic investigation following endoscopic marking made it possible to identify the responsible lesion and resect a minimum length of the small bowel.

## Data Availability

Data sharing is not applicable to this article as no datasets were generated or analyzed.
